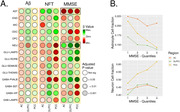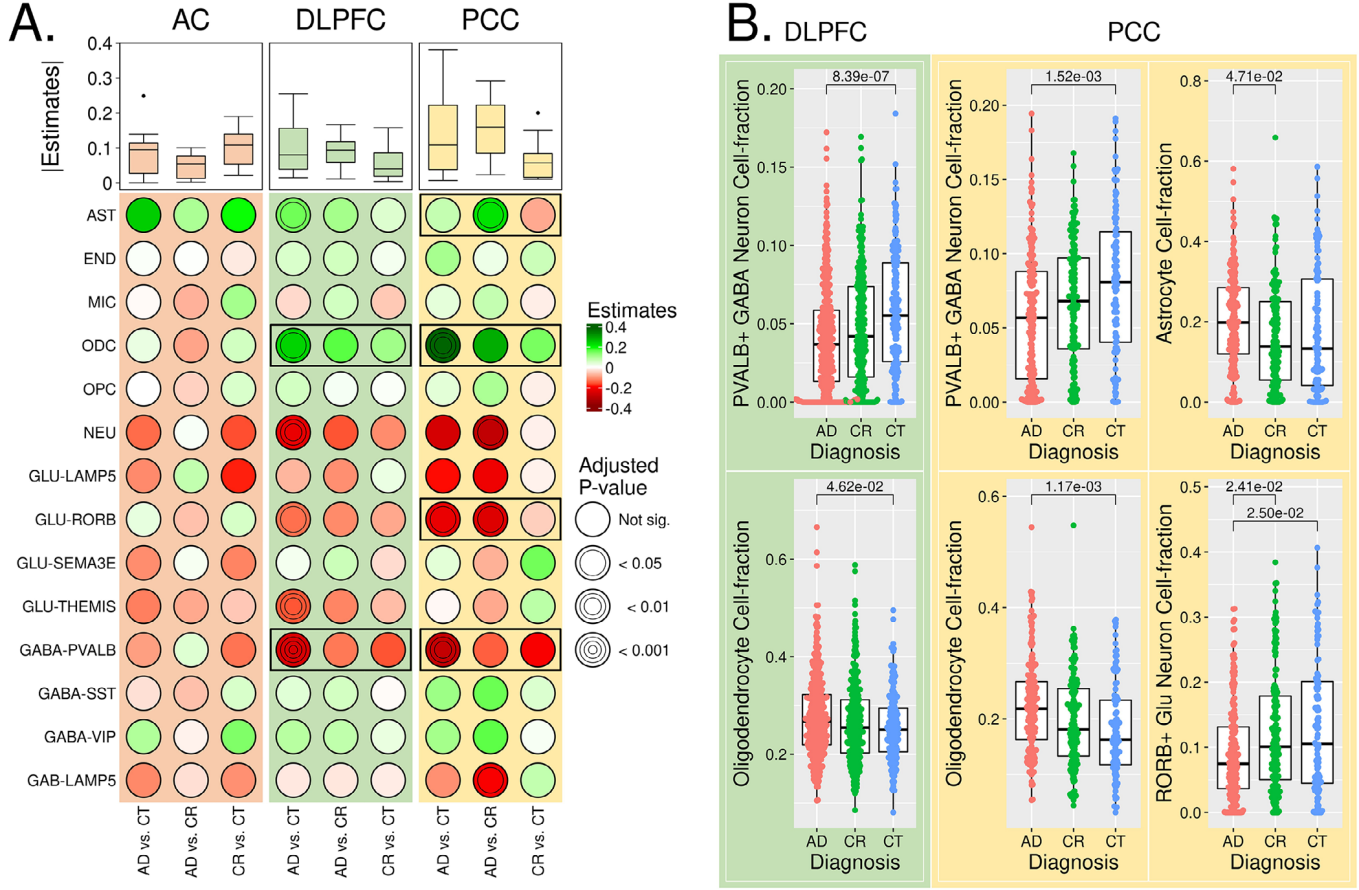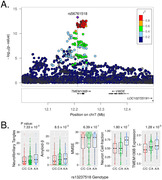# Cognitive resilience to Alzheimer’s disease characterized by cell‐type abundance estimates

**DOI:** 10.1002/alz.091390

**Published:** 2025-01-03

**Authors:** Nicholas K O'Neill, Thor D. Stein, Oluwatosin A Olayinka, Jenny A Empawi, Junming Hu, Tong Tong, Xiaoling Zhang, Lindsay A. Farrer

**Affiliations:** ^1^ Boston University, Boston, MA USA; ^2^ Boston University Chobanian & Avedisian School of Medicine, Boston, MA USA; ^3^ VA Boston Healthcare System, Boston, MA USA; ^4^ VA Bedford Healthcare System, Bedford, MA USA; ^5^ Department of Medicine (Biomedical Genetics), Boston University Chobanian & Avedisian School of Medicine, Boston, MA USA; ^6^ Boston University School of Public Health, Boston, MA USA

## Abstract

**Background:**

Some subjects exhibit AD pathology but remain cognitively intact. This resilience has been associated with cell‐type abundance changes, particularly in neurons. We investigated the molecular basis of cognitive resilience by deconvoluting bulk RNA sequencing (RNA‐seq) data into multiple brain cell types derived from three brain regions.

**Methods:**

Cell‐type abundance in human brain bulk RNA‐seq data from 2,457 samples among three brain regions (744 anterior caudate (AC), 1,141 dorsolateral prefrontal cortex (DLPFC), and 572 posterior cingulate cortex (PCC)) obtained from ROSMAP Study participants (434 AD cases, 318 cognitively resistant (CR) AD cases and 188 controls (CT) was estimated by deconvolution using snRNA‐seq cell‐type signatures and DeTREM software. Abundance of six major cell types and eight neuronal subtypes were compared between pairs of diagnostic groups and for three AD clinicopathological traits (amyloid‐β burden and neurofibrillary tangles quantified by immunohistochemistry, and MMSE score) using a generalized linear model including covariates for cell‐fraction, age, sex, and post‐mortem interval. We also conducted a genome‐wide association study (GWAS) for abundance of each cell‐type and brain region using a model that included a polygenic random effect, genetic relationship matrix, and the covariates listed above.

**Results:**

Parvalbumin positive (PVALB+) inhibitory neuron abundance was negatively associated with cognitive status and tau pathology in DLPFC and PCC (all comparisons p_adj_<0.001, Figure 1) and the most reduced neuronal subtype in AD cases compared to controls in the DLPFC (p_adj_ = 8.4 × 10^‐7^) and PCC (p_adj_ = 0.0015) regions (Figure 2). The proportions of astrocytes and RORB expressing neurons in the PCC were significantly increased (p_adj_ = 0.047) and decreased (p_adj_ = 0.024), respectively, in CR AD cases compared to non‐CR AD cases. We also identified a genome‐wide significant association of neuron abundance with *TMEM106B* SNP rs13237518 in PCC (p = 6.08 × 10^‐12^). This variant was also associated with levels of amyloid‐β (p = 0.0085) and tangles (p = 0.0073) in the same region (Figure 3).

**Conclusion:**

High abundance of PVALB+ neurons and lack of astrocytosis may be markers of cognitive resilience among persons with a pathological diagnosis of AD. Variants in *TMEM106B* are associated with neuron abundance and, hence, may specifically influence resilience to cognitive decline independent of AD pathology.